# Interactions between αv-Integrin and HER2 and Their Role in the Invasive Phenotype of Breast Cancer Cells In Vitro and in Rat Brain

**DOI:** 10.1371/journal.pone.0131842

**Published:** 2015-07-29

**Authors:** Sangeet Lal, Cymon Kersch, Kathleen A. Beeson, Y. Jeffrey Wu, Leslie L. Muldoon, Edward A. Neuwelt

**Affiliations:** 1 Department of Neurology, Oregon Health & Sciences University, Portland, Oregon, United States of America; 2 Department of Cell, Developmental and Cancer Biology, Oregon Health & Sciences University, Portland, Oregon, United States of America; 3 Department of Neurosurgery, Oregon Health & Sciences University, Portland, Oregon, United States of America; 4 Veterans Administration Medical Center (EAN), Portland, Oregon, United States of America; Thomas Jefferson University, UNITED STATES

## Abstract

**Background:**

We tested the hypothesis that αv-integrin and the human epidermal growth factor receptor type 2 (HER2) interact with each other in brain trophic metastatic breast cancer cells and influence their invasive phenotype.

**Methods:**

Clones of MDA-MB231BR human breast cancer cells with stable knock down of αv-integrin in combination with high or low levels of HER2 were created. The interactions of these two proteins and their combined effect on cell migration and invasion were investigated in vitro and in vivo.

**Results:**

Knockdown of αv-integrin in MDA-MB231BR clones altered the actin cytoskeleton and cell morphology. HER2 co-precipitated with αv-integrin in three breast cancer cell lines in vitro, suggesting they complex in cells. Knockdown of αv-integrin altered HER2 localization from its normal membrane position to a predominantly lysosomal localization. When αv-integrin expression was decreased by 69–93% in HER2-expressing cells, cellular motility was significantly reduced. Deficiency of both αv-integrin and HER2 decreased cellular migration and invasion by almost 90% compared to cells expressing both proteins (P<0.01). After intracerebral inoculation, cells expressing high levels of both αv-integrin and HER2 showed a diffusely infiltrative tumor phenotype, while cells deficient in αv-integrin and/or HER2 showed a compact tumor growth phenotype. In the αv-integrin positive/HER2 positive tumors, infiltrative growth was 57.2 ± 19% of tumor volume, compared to only 5.8 ± 6.1% infiltration in the double deficient tumor cells.

**Conclusions:**

αv-integrin interacts with HER2 in breast cancer cells and may regulate HER2 localization. The combined impacts of αv-integrin and HER2 influence the invasive phenotype of breast cancer cells. Targeting αv-integrin in HER2-positive breast cancer may slow growth and decrease infiltration in the normal brain.

## Introduction

Breast cancer is the most common neoplasm in women and ranks as the second most common malignancy to form brain metastases, which are associated with poor prognosis and rapid mortality [1]. There is still limited knowledge of the biomolecular factors and mechanisms controlling invasion of systemic cancer cells into the brain, and few options available for the prevention or treatment of brain metastases [2]. The process of metastasis requires detachment of cells from the primary tumor, invasion of the extracellular matrix (ECM), travel through the circulatory system, extravasation with adhesion to endothelial cells, and invasion and survival in the foreign microenvironment [3].

Cancer cells depend on members of the integrin family of transmembrane receptors, essential mediators of cell-ECM and cell-cell interactions, for multiple steps in the metastatic cascade [4–6]. Integrins are obligate αβ dimers, from a pool of 18 α and 8 β subunits, forming 24 known heterodimers. The αv-integrins are frequently overexpressed in metastases [7–10] appear to be important in the survival, proliferation, migration and invasion of cancer cells [4–6]. Activation of αvβ_3_-integrin promotes tumor angiogenesis and metastatic growth in mouse brain [11], while transcriptional silencing of these integrins with MYC decreases migration and invasion of breast cancer cells in vitro and in vivo [12]. In preclinical models, targeting αv-integrin with the monoclonal antibody intetumumab or αvβ3- and αvβ5-integrins with the cyclic peptide cilengitide has shown anti-tumor effects as well as metastasis prevention activity [13–15]. However, in clinical trials, intetumumab and cilengitide have demonstrated minimal therapeutic efficacy inducing tumor cell death in metastases [16–18]. The inadequacies of current therapy emphasize the need to precisely understand the tumor-specific biology and signaling so that suitable biomarkers and therapeutic targets can be identified.

Cancer cell migration, invasion and proliferation are driven by a dynamic and complex array of highly integrated signaling cascades [19]. The human epidermal growth factor receptor 2 (HER2), also known as ErbB2, an orphan receptor tyrosine kinase, is implicated in enhanced breast cancer cell proliferation and aggressive tumorigenic behavior [20]. Malignancies with HER2 overexpression show increased brain metastatic outgrowth in preclinical models [21] and a high incidence of brain metastases clinically, with up to 30% of patients developing central nervous system lesions [22,23]. However, it is poorly understood how HER2-overexpressing cells gain an invasive or metastatic phenotype that requires dynamic remodeling of cell adhesion and actin cytoskeletal assembly and navigation of the ECM [3,23]. Physical interactions between integrins and various growth factor receptors and crosstalk between these signaling systems have been reported in normal and pathological conditions, including cancer [24–26] and may alter the effect of HER2-targeted therapies [27,28]. Thus, understanding how integrins relate to other key drivers of cancer metastasis is essential and may provide pharmacologic targets for simultaneous inhibition of multiple molecular pathways.

In this study, we evaluated the interaction of αv-integrin and HER2 using a brain-trophic breast cancer cell line (MDA-MB231BR-HER2) [21] and two non-transformed HER2-positive breast cancer cell lines. Our results suggest that αv-integrin complexes with and regulates the cellular localization of HER2. Additionally, the concomitant presence of both proteins increases the migration and invasion of breast cancer cells in vitro and in the brain microenvironment.

## Materials and Methods

### Cell lines and preparation of stable knockdown clones

The brain-trophic human breast cancer cells transfected to express high HER2 (MDA-MB231BR-HER2, herein termed MM2BH cells) were obtained from Dr. Patricia Steeg (NCI, MD) and cultured in DMEM (Corning Life Sciences—Mediatech, Manassas VA) supplemented with 20% fetal bovine serum (Innovative research, Novi MI), 20mM HEPES (Sigma-Aldrich, St. Louis MO) and 350mM Zeocin (Life Technologies, Eugene OR). Cells were transduced with one of two distinct αv-integrin sequence-specific lentiviral shRNAi constructs (TRCN-768 and TRCN-769) or a scrambled control shRNA (TRC1) derived from the MISSION library of Sigma-Aldrich. After 48h, transduced cells were seeded in 96-well plate with limited dilution per well and cultured in the presence of 1μg/ml puromycin. Stably transduced clones were selected and expanded from individual wells. The expression of αv-integrin as well as HER2 in selected clones was verified by western blotting of whole cell lysates and by flow cytometry. Five MM2BH clones with high or low expression of αv-integrin and high or low HER2 expression were chosen for study: (a) αv+H2+, (b) αv+H2-, (c) αv-H2+sR1 (shRNA TRCN-769), (d) αv-H2+sR2 (shRNA TRCN-768), and (e) αv-H2-. Knockdown clones were maintained in 0.5 μg/ml puromycin (Life Technologies, Eugene OR). SKBR3 and HCC1954, two non-transformed high HER2 breast cancer cell lines, were obtained from ATCC (Manassas VA), and cultured in McCoys Medium (Corning Life Sciences—Mediatech, Manassas VA) and RPMI1640 (Lonza BioWhittaker, Allendale NJ) respectively, supplemented with10% fetal bovine serum.

### Immunofluorescence

Cells grown on glass coverslips were fixed with 4% paraformaldehyde (Tousimis, Bethesda MD) at room temperature (RT) or methanol at -20C. Formaldehyde-fixed cells were permeabilized with 0.25% TritonX-100 (Sigma, St. Louis MO) and blocked in 5% bovine serum albumin (Sigma, St. Louis MO). Cells were incubated overnight in primary antibodies (1:200) and fluorophore-conjugated species-specific secondary antibody was used at 1:600 dilution for 1-2h at RT. Antibodies used in this study are as follows; αv-integrin (Q20-R; Santa Cruz Biotechnology, Dallas TX), HER2 (clone 29D8, Cell Signaling Technology, Beverly MA), LAMP2 (Southern Biotech, Birmingham AL), Alexa Fluor-conjugated secondary antibodies were purchased from Life Technologies (Eugene OR). F-Actin was stained with rhodamine phalloidine (1:200; Life Technologies, Eugene OR) and Hoechst (1:5000) was used to co-stain nuclei, and coverslips were mounted on microscope slides with ProLong Gold antifade reagent (Life Technologies, Eugene OR). High-resolution images were captured at 40x magnification using a Zeiss Apotome microscope (Carl Zeiss Microscopy, Munich Germany) at the OHSU Advanced Light Microscopy core facility.

### Western blotting and Immunoprecipitation

Cells were harvested using TrypLE Express (Life Technologies, OR) and suspended in cold RIPA buffer (50mM Tris-Cl, 150mM NaCl, 1% Triton X-100, 0.5% Na-deoxycholate, 0.1% SDS) supplemented with 1x Halt protease inhibitor cocktail (Thermo Scientific, Waltham MA). After 15min on ice, the suspension was centrifuged at 4°C to clear insoluble debris from supernatant lysate. Protein concentration was measured with the BCA assay kit (Thermo Scientific, Waltham MA) and the western blotting was performed using SDS-PAGE. The intensity of individual protein bands on immunoblots was quantified using UN-SCAN-IT gel software 6.1 (Silk Scientific., Orem UT) and normalized with that of β-actin (Sigma-Aldrich, St. Louis MO) loading control. For co-immunoprecipitation assays, modified lysis buffer (50mM Tris-Cl pH 7.5, 150mM NaCl, 1% NP-40, 0.5% Na-deoxycholate, 1mM EDTA and 1mM EGTA) supplemented with 1x Halt protease and phosphatase inhibitor cocktail, was used. After 30 min incubation in the lysis buffer on ice, the cell suspension was passed through a 21-gauge needle to disrupt genomic DNA and centrifuged to clarify unsolubilized proteins. 500μg cell lysate was incubated overnight with 5μg antibody against HER2 or αv-integrin. ProteinA-conjugated agarose beads (Thermo Scientific, Waltham MA) were added and incubation was continued for additional 2-3h. The immunocomplex was separated from unbound lysate by centrifugation and then pulled down proteins were eluted by boiling at 95C for 5 min in Lamaelli buffer (Bio-Rad, Hercules CA).

### Live Cell Flow Cytometry

Cells were harvested using TrypLE Express (Life Technologies, OR) and suspended in blocking buffer (3% BSA in PBS pH 7.4) as a single-cell suspension. Cell surface protein expression was evaluated with the following antibodies: anti-HER2/neu-APC (BD340554, BD Biosciences; 2.5uL per reaction), anti-αv-integrin (Q20-R, Santa Cruz Biotechnology; 1:200), and Alexa Fluor 647 conjugated secondary antibody (Life Technologies; 1:500) was used for αv-integrins. Cells (10^6^ per antibody) were incubated with primary or direct conjugated antibodies for 45 min at 4C, then with secondary antibodies for 30 min at 4C. Cells were washed and resuspended in FACS buffer (PBS with10% FBS and 0.1% NaN_3_), and assessed within 1 h of antibody staining using the FACSCanto II flow cytometer (BD Biosciences, San Jose CA) at the OHSU flow cytometry core. Analyses were completed using FlowJo software (http://www.flowjo.com/).

### Proliferation, Migration and Invasion in vitro

Cell growth was determined by serial measurement of the number of viable cells after seeding of 2500 cells in opaque-walled 96-well plates, using the Cell-Titer Glo luminescent viability assay kit (Promega, Madison WI). Luminescence intensity was quantified using a BioTek Flx800 plate reader (Winooski, VT). The migration and invasion of MM2BH clones were examined using transwell inserts (8μm pores; Corning, Manassas VA). Uncoated inserts were used in migration, and for the invasion assay, the upper chamber was coated with 30μg of Matrigel (BD Bioscience, San Jose CA). 50,000 cells, suspended in serum-free media, were added to the upper chamber and 10% serum was used as the chemoattractant in lower wells. Migration was continued for 8h and invasion for 64h after cell seeding. Cells were removed from the upper chamber with cotton swabs and cells that migrated on the bottom of the membrane were fixed with chilled methanol for 15min. Subsequently, cells were stained in 1:5000 Hoechst solution (Life Technologies, Eugene OR) for 30min. The membranes were excised and mounted cell-side down on microscope slides using ProLong Gold antifade reagent (Life Technologies, Eugene OR). Ten random fields were imaged at 5x objective magnification on the Zeiss Apotome microscope and the number of cells per field was quantified using the ImageJ software (NIH, Bethesda MD).

### In vivo assessment of cell growth and infiltration

The care and use of animals was approved by the Institutional Animal Care and Use Committee and supervised by the OHSU Department of Comparative Medicine. Female nude rats (rnu/rnu, 200–250g) were anesthetized with intraperitonial injection of ketamine (60 mg/kg) and diazepam (7.5 mg/kg). Buprenorphine (0.1 mg/kg) was applied subcutaneously for analgesia. The head was shaved and the rat placed in a stereotactic frame (David Kopf Instruments, Tejunga CA). A 2-mm diameter hole was drilled in the skull and a 27-gauge needle was lowered slowly into the right caudate putamen using coordinates (bregma = 0; lateral = -0.31, vertical = -0.65). Animals were randomized and inoculated with 1.5x 10^6^ of each MM2BH cell clone: (a) αv+H2+, (b) αv+H2-, (c) αv-H2+sR1, (d) αv-H2+sR2, and (e) αv-H2- (n = 6–7 rats per group). Animals were monitored daily by Department of Comparative Medicine personnel and by research staff. The predetermined end point was 5 weeks after tumor implantation, and no animals showed distress or weight loss requiring early euthanasia. Euthanasia was performed by CO_2_ asphyxiation. Brains were fixed in formalin and 100μm thick coronal slices were sectioned using a vibratome (HM650V, Thermo Scientific, Waltham MA). Immunohistochemistry was performed on every sixth section by staining for human mitochondrial antigen (Abcam, Cambridge MA). Total tumor area was manually outlined on scans of all stained sections by a blinded researcher and assessed using ImageJ software (NIH). Tumor volume was determined by multiplying the sum of measured areas by the section thickness and section separation (sum [mm2] x0.1mm x6). The area of tumor visually judged to match the “infiltrative” phenotype was manually outlined in 2–6 central sections and the area occupied by infiltrating tumor cells was determined as a percentage of total tumor area. 1-2 rats in each group showed incorrect tumor placement (ventricle or base of brain) or excessive post-mortem autolysis, and these tumors were not included in the analysis of the infiltrative phenotype.

### Statistical analysis

In vitro studies were performed at least 3X with 3–5 replicates per group. Single time point data were compared by Student’s *t*-test and multiple time points were assessed with repeated measures ANOVA. For the animal study, no power calculations were made *a priori* or *post hoc*. Tumor volume (total and infiltrative) was compared using ANOVA to determine overall significance and Student’s *t*-test for comparison of individual groups. Analyses were performed with Microsoft Excel and Graphpad Prism software.

## Results

**Fig 1:**
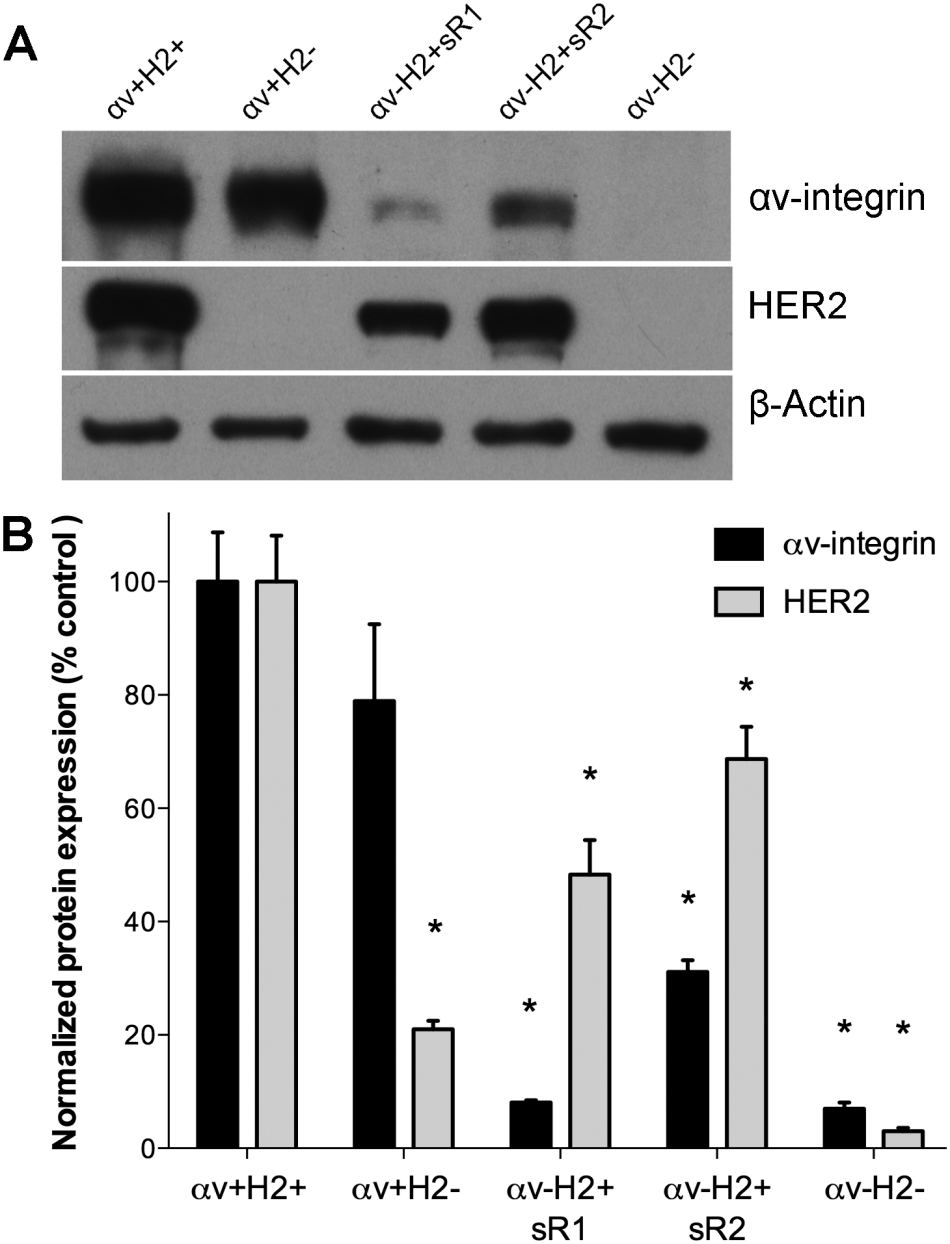
Integrin and HER2 expression in brain-trophic breast cancer cell clones. MM2BH breast cancer cells, previously transfected to overexpress HER2, were transduced with lentiviral shRNAs (sR), scrambled or specific to αv-integrin, to create stable knockdown cell lines in combination with high or low HER2 protein. (A) Immunoblotting of protein expression in MM2BH lines with αv integrin knockdown in combination with high or low HER2 expression. (B) Relative expression of αv integrin and HER2 in MM2BH clones quantified by densitometry of immunoblots from three total cell lysates prepared at different culture passages. The band intensity was normalized to β-actin as a loading control. Error bars show SEM and * indicates values P<0.05 in comparison to control cells.

Breast cancer cell clones with stable 93% and 69% knockdown of αv-integrin were created in a human breast cancer cell line MDA-MB231BR, herein referred to as MM2BH cells, using two independent shRNA vectors. Since MM2BH cells heterogeneously express HER2 protein, each knockdown clone was examined for HER2 and we identified clonal cell lines with different combinations of αv-integrin and HER2 protein levels. Sixty clones were evaluated, and a set of clones with relatively high or low expression of each protein was selected: (a) αv+H2+ (high αv integrin, high HER2) and (b) αv+H2- (high αv, low HER2) were both generated with the scrambled control shRNA; (c) αv-H2+sR1 (low αv, high HER2 with shRNA TRCN-769), (d) αv-H2+sR2 (low αv, high HER2 with shRNA TRCN-768), and (e) αv-H2- (low αv, low HER2, with the αv-integrin shRNA TRCN-769). Fig 1A shows a representative immunoblot, while Fig 1B indicates protein levels quantified from three independent blots. The level of HER2 in the integrin knockdown clones, αv-H2+sR1 and αv-H2+sR2, was 52% and 31% lower than the αv+H2+ control cells, respectively (P<0.05).

**Fig 2:**
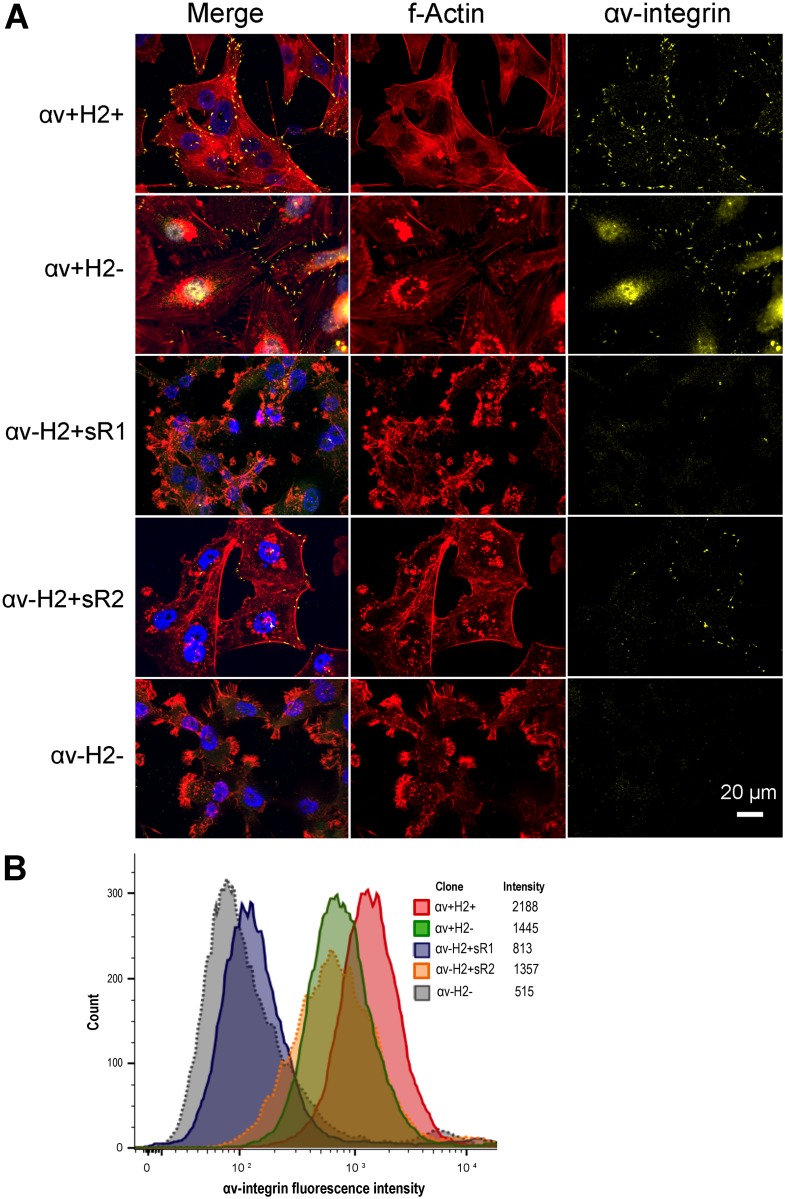
Characterization of cell morphology, actin cytoskeleton and focal adhesions in breast cancer cells. MM2BH clones grown on glass coverslips were stained for αv-integrin and actin filaments. The images are representative of three independent experiments. Yellow: αv-integrin, red: f-actin, blue: nucleus. Scale bar = 20μm. (B) Flow cytometry analysis of cell surface αv-integrin protein expression in live MM2BH clones.

We used immunofluorescence staining to assess the impact of αv-integrin knockdown on the cytoskeleton, cellular morphology and focal adhesion in MM2BH clones (Fig 2A). Control αv+H2+ cells showed a spreading and adhesive phenotype with prominent actin fibers and αv-integrin primarily localized to cell surface focal adhesions on cell membrane protrusions. Knockdown of αv-integrin in the αv-H2+sR1 and αv-H2- cells resulted in reduced cell spreading, extensive membrane ruffling with spike-like protrusions and few focal adhesions (Fig 2A). The effect of αv-integrin knockdown on cellular morphology was most pronounced in the double deficient αv-H2- MM2BH cells. Live cell flow cytometry to further assess cell surface αv-integrin protein levels showed lower mean florescence intensities confirming decreased surface αv-integrin, particularly in the αv-H2+sR1 and αv-H2- cells (Fig 2B).

**Fig 3:**
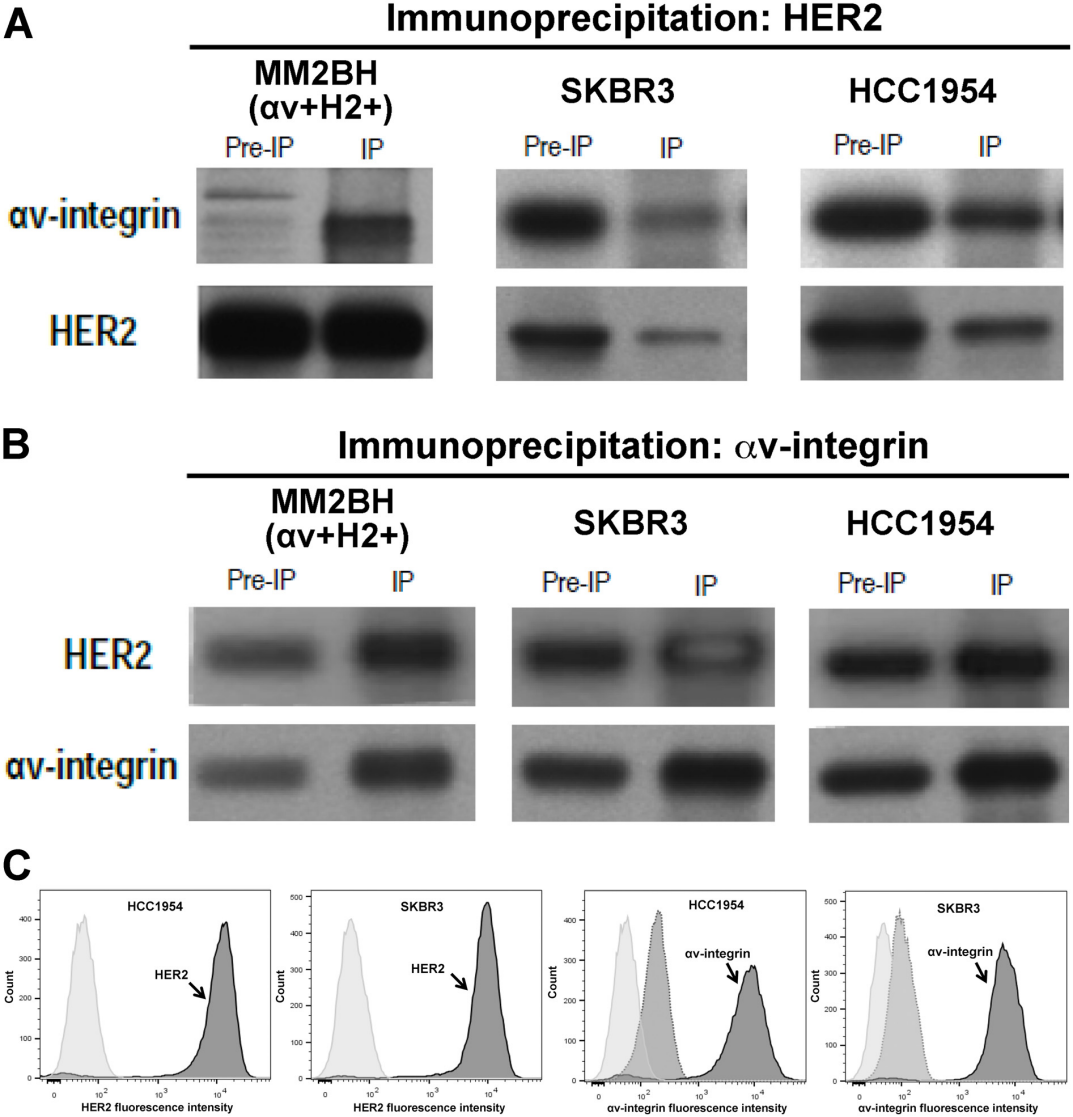
Co-immunoprecipitation of αv-integrin and HER2 proteins in breast cancer cells. Lysates from the SKBR3, HCC1954, and αv+H2+ MM2BH cells was incubated separately with antibodies against HER2 (A) and αv-integrin (B). Components of the immunocomplex precipitate were analyzed by Western blotting for αv-integrin and HER2 proteins. Protein levels are shown in whole lysate (pre-IP), and the immunoprecipated protein complex (IP). (C) Flow cytometry confirmation of HER2 and αv-integrin expression (arrows) in HCC1954 and SKBR3 cells. Light gray peak is unstained control and medium grey is secondary antibody only control.

Co-immunoprecipitation was used to explore the physical interaction between HER2 and αv-integrin proteins in the αv+H2+ cells as well as two additional non-transformed human breast cancer cell lines, HCC1954 and SKBR3. αv-integrin co-precipitated with HER2 under non-denaturing conditions in all 3 cell lines (Fig 3A). Similarly, HER2 protein was immunoprecipitated by using anti-αv-integrin antibodies (Fig 3B). We confirmed that HCC1954 and SKBR3 cells express both HER2 and αv-integrin on the cell surface using flow cytometry (Fig 3C).

**Fig 4:**
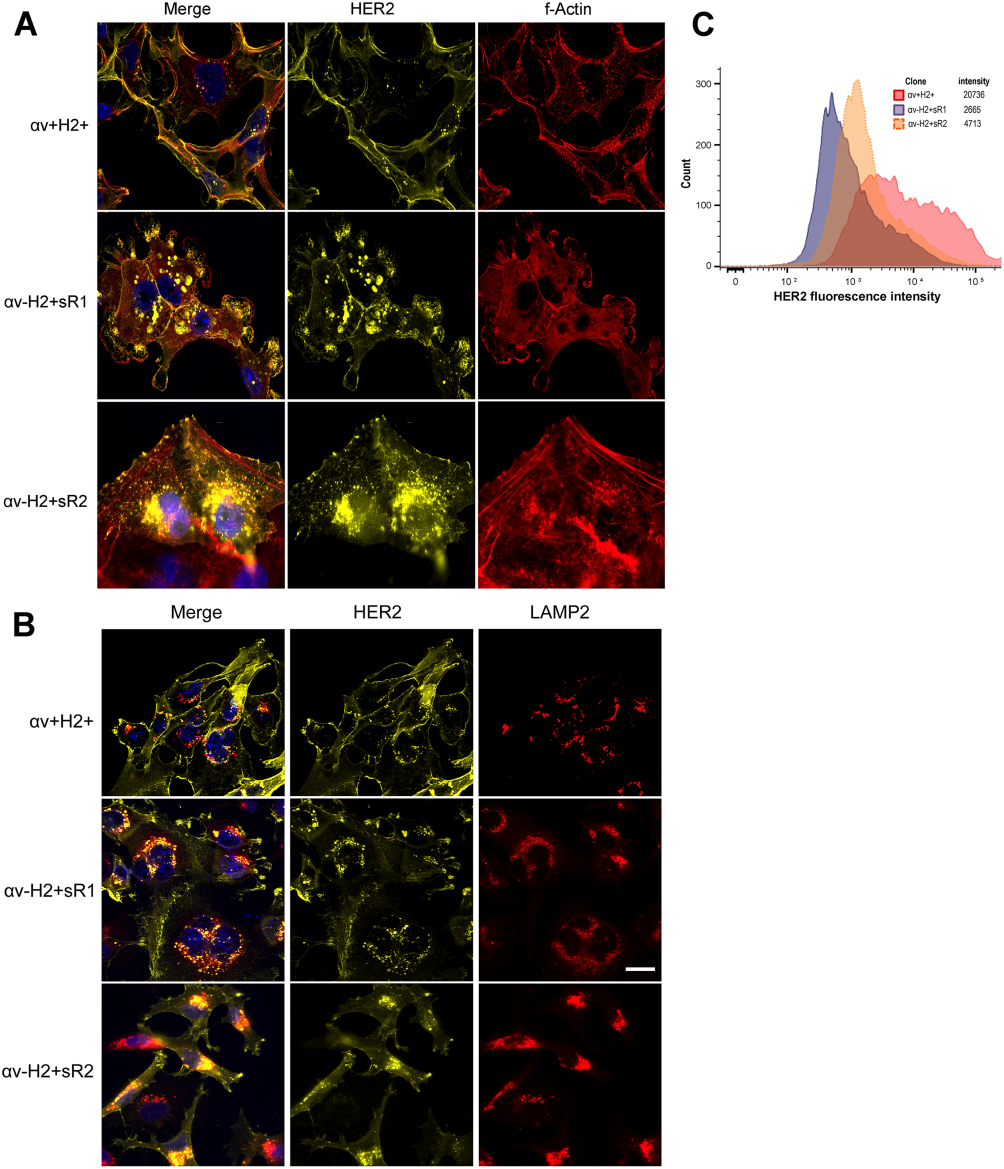
localization of HER2 in αv-integrin knockdown breast cancer cells. MM2BH cell clones grown on glass coverslips were fixed, permeabilized and immunostained. (A) Deficiency of αv-integrin results in increased cytoplasmic localization of HER2 receptors. Yellow: HER2, using an antibody specific for the HER2 intracellular domain, red: f-actin, blue: nucleus. (B) HER2 co-localizes with lysosomes in αv-integrin knockdown cells. Yellow: HER2, red: lysosome-associated membrane protein2 (LAMP2), blue: nucleus. The images are representative of three independent experiments. Scale bar = 20μm. (C) Flow cytometry analysis of surface HER2 protein expression in live MM2BH clones.

We next examined the role of αv-integrin on the cellular distribution of HER2 protein. In the MM2BH αv+H2+control cells, HER2 was uniformly distributed on the plasma membrane with scarce subcellular staining. In contrast, the αv-integrin knockdown clones showed increased levels of cytoplasmic localization of HER2 in vesicles, predominantly in the perinuclear space (Fig 4A). Minimal HER2 immunofluorescence was sporadically observed on the cell membrane of αv-integrin knockdown cells, while intense globular HER2 staining was observed in the sub-membrane space. Because the HER2 immunostaining in both αv-integrin knockdown clones was suggestive of lysosomal or late endosomal localization, we assessed its proximity to the lysosome-associated membrane protein 2 (LAMP2). Immunofluorescence showed that the cytoplasmic staining of HER2 strongly co-localized with LAMP2-positive lysosomal vesicles in the knockdown cells (Fig 4B). To confirm the decreased surface localization of HER2 in these clones, live cell flow cytometry was performed. Results show lower mean florescence intensities in the αv-H2+ clones compared to the control αv+H2+ clone (Fig 4C).

**Fig 5:**
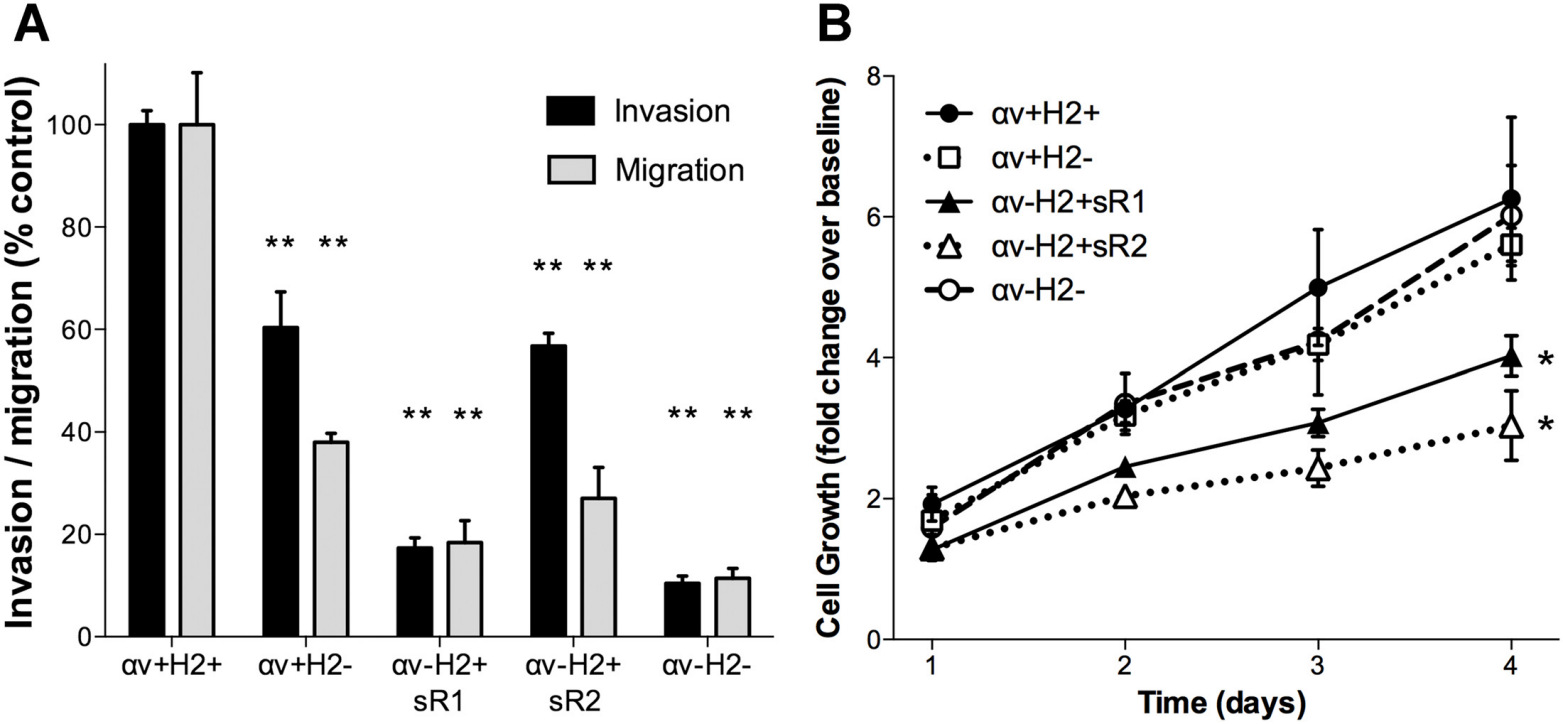
In vitro cell migration and proliferation of breast cancer cell clones. (A) The motility of MM2BH cell lines was examined in transwell assays in the absence (migration, 8h) and presence of Matrigel extracellular matrix (invasion, 64h) using FBS as chemoattractant. The graph represents average motility from six replicates from independent experiments. Bars show mean ± SEM. (B) Cell proliferation was measured every 24h with the Cell-Titer Glo luminescence assay. The graph shows average fold increase from eight replicate wells from separate experiments. Data are presented as mean ± SEM, *P<0.05, ** P<0.001 in comparison to control cells.

To assess the impact of αv-integrin and HER2 deficiency on in vitro migration and invasion of MM2BH cells transwell migration and invasion assays were performed. The αv-integrin knockdown clones showed significant inhibition of motility compared to control cells; over 80% lower migration and invasion was observed for αv-H2+sR1 cells and 73% and 46% decrease in migration and invasion respectively, for αv-H2+sR2 cells (Fig 5A, P<0.01). The αv+H2- cells, with 21% lower αv-integrin content than control cells, also showed 60% lower migration and 40% lower invasion. The most pronounced decrease in migration and invasion, 90% lower than control cells, was recorded for αv-H2- cells (P<0.01). To determine whether altered protein expression affected cell proliferation, the growth of each clone was measured by a luminescence-based assay. The knockdown of αv-integrin, with high HER2, significantly inhibited cell growth in the two independent clones compared to control (Fig 5B, P<0.05). In contrast, cells lacking only HER2, or deficient in both αv-integrin and HER2 (αv-H2- cells), proliferated at the same rate as control cells.

**Fig 6:**
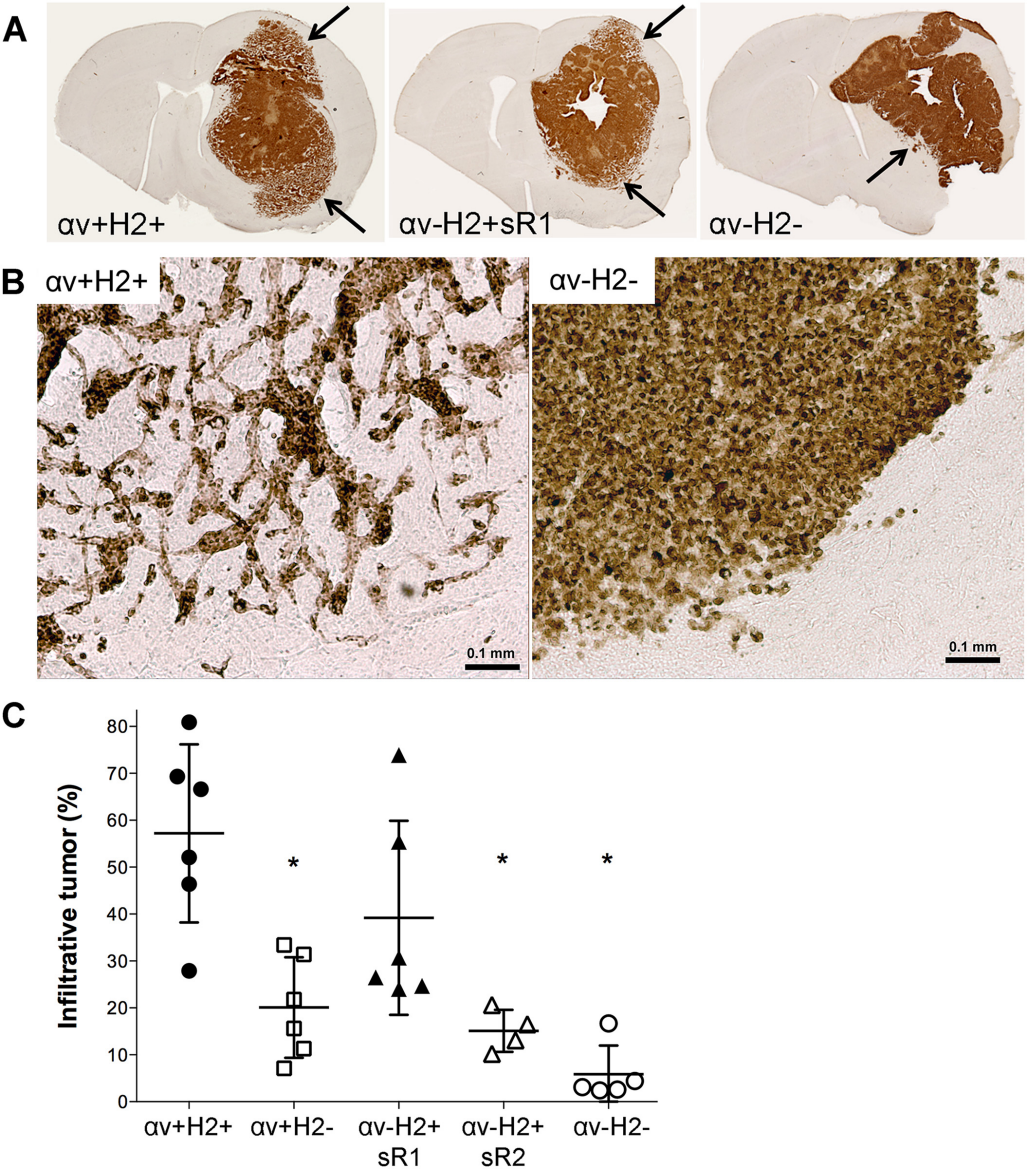
In vivo invasive growth phenotype in αv-integrin and HER2 knockdown breast cancer cells. Brain tumors of MM2BH clones were assessed by immunohistochemistry for human mitochondrial antigen 5-weeks after intracerebral implantation. (A) Representative sections from αv+H2+, αv-H2+sR1, and αv-H2- tumors. Arrows indicate regions of tumor infiltration. (B) Brain tumor margin showing the infiltrative phenotype in αv+H2+ and αv-H2- tumors. (C) Quantification of the percent of tumor showing the infiltrative phenotype in each tumor type. Each point indicates the mean value from 2–6 central sections from each rat brain. *p<0.05 compared to control.

To evaluate the influence of αv-integrin and HER2 protein expression levels on the proliferation and dispersal of breast cancer cells in the brain microenvironment, we assessed the growth characteristics of each MM2BH clone after intracerebral implantation in athymic nude rats. Xenografts of all five clones grew over the 5-week assessment period. Tumor volume was highly variable within each cell clone, with small and large tumors in each group, so there was no difference in tumor volume between groups (overall mean 85.2 ± 58.6 mm^3^). Sections from αv+H2+, αv-H2+sR1, and αv-H2- brain tumors (Fig 6A) show markedly different patterns of brain infiltration (arrows). Xenografts expressing both αv-integrin and HER2 have a central solid tumor mass surrounded by an extensive region of tumor-infiltrated brain (Fig 6B). In contrast, the double deficient cells display a compact tumor mass without infiltration and the single deficient clone shows an intermediate phenotype. A blinded investigator quantified the percentage of tumor volume that was a solid mass versus the diffuse growth pattern (Fig 6C). In αv+H2+ tumors, 57.2 ± 19.0% of the tumor was infiltrative, which was significantly different from the αv+H2- and αv-H2+sR2 tumors. In the double deficient clone αv-H2-, infiltration was 5.8 ± 6.1% of the tumor mass, which was significantly less than all other groups (P<0.05).

## Discussion

The diagnosis of brain metastasis is associated with extremely poor prognosis, yet current therapies are limited to radiation and surgical interventions and prevention strategies are restricted to whole brain radiation [2]. Improving pharmacologic therapies would be extremely beneficial, but requires a better understanding of the unique biomolecular properties of brain-trophic tumor cells. A complete picture of how cancer cells recognize, bind to, and migrate through brain vasculature, infiltrate into brain parenchyma, and thrive in the brain microenvironment remains unclear [3,19,23].

Individually, the HER2 receptor tyrosine kinase and integrin cell adhesion proteins are known factors in metastasis. HER2 accentuates the metastatic phenotype and brain organotropism in breast cancers, increasing brain metastasis in preclinical models [21] and in patients [22,23]. However, despite the prevalence of HER2 in brain metastases, anti-HER2 agents such as lapatinib and trastuzumab have not yet found a role in prevention or treatment of this disease [23]. Similarly, αv-integrin has emerged as a negative diagnostic biomarker [7–10]. Targeting αv-integrin has both therapeutic and preventive activity in preclinical models [13–15], but these benefits have not transferred to the clinical setting [16–18].

### Interactions of αv-integrin and HER2

In this study, we investigated the relationship between αv-integrin and HER2 and their roles in the invasive phenotype of breast cancer cells. Multiple reports document direct interactions between various integrins and HER2, suggesting that interactions between these proteins may be important in certain metastatic cancers [4,25]. In breast cancer cells, α3β1 integrin regulates dimerization of HER2 to the active form [24], and β4-integrin enhances HER2 activity and stimulates mammary cell tumorigenesis [29]. Additionally, α6β4 integrin interactions with HER2 are increase cell motility and survival [30], and inhibition of this heterodimer blocks HER2 signaling and growth of orthotopic xenografts [27].

We observed co-immunoprecipitation of αv-integrin and HER2 (Fig 3) suggesting there is a physical interaction between αv-integrin and HER2 or a protein complex that includes both proteins. Co-immunoprecipitation is a standard technique for assessing integrin protein interactions [11,12,25], including with HER2 [26]. The parental cell line for the MM2BH clones was derived from triple-negative breast cancer cells (MDA-MB231) transfected to overexpress HER2 [21]. To confirm that the interactions between HER2 and αv-integrin were a generalized phenomenon, we showed similar co-immunoprecipitation using two nontransfected human breast cancer cell lines (HCC1954 and SK-BR3) with high αv-integrin and HER2 expression. In all cells, strong immunoblot signals showed HER2 co-precipitated when the αv-integrin antibody was used for immunoprecipitation.

We evaluated if αv-integrin expression could modulate HER2 localization. Immunofluorescent images reveal increased localization of HER2 in cytoplasmic vesicles, particularly lysosomes, and under the membrane at the cell periphery in αv-integrin knockdown cells (Fig 4). This finding suggests a role of αv-integrin in either trafficking or stabilization of HER2 receptors on the cell membrane. While the particular cellular mechanisms underlying this localization role are yet to be determined, possible explanations include a novel contribution of integrins in the retention model for HER2, or influence of integrins on HER2 membrane trafficking and recycling. The retention model requires binding of HER2 with actin-associated PDZ-domain proteins such as erbin and/or LIN-7, for the proper maintenance of HER2 at the plasma membrane [31,32]. A functional crosstalk between E-cadherin and αv-integrin proteins has been reported in breast carcinoma cells [33]. Considering that MM2BH cells with low αv-integrin (αv-H2+sR1) have poorly spread morphology with extensive membrane ruffling and sparse focal adhesions (Fig 2), low levels of αv-integrin could potentially interfere with the formation of actin-associated proteins assembly including E-cadherins, PDZ-domain proteins and LIN-7, resulting in impaired retention of HER2 proteins on the cell membrane. Alternately, as growth factor receptors on the membrane are subjected to endosomal trafficking pathways, there may be a role for integrins in regulating HER2 recycling or endocytotic pathways leading to lysosomal degradation [31,32]. Unlike other members of the epidermal growth factor receptor family, HER2 internalization is slow and is at least somewhat resistant to membrane down-regulation [31,32], which makes the finding of lowered levels of membrane localized HER2 even more intriguing. Our data suggest that αv-integrin may be a modulate HER2 membrane stability and localization, and thus, altering αv-integrin levels could affect HER2 function and exposure to targeted therapies.

HER2 expression may also impact αv-integrin cell surface localization, as demonstrated by both flow cytometry (Fig 1C) and immunofluorescence staining (Fig 2). Surface expression of integrins involves complex, dynamic, and tightly regulated trafficking and recycling processes linked to the activation and ligand bound state of integrins [34]. Integrin recycling depends on activation state [35] and influences signal transduction [36]. In metastasizing cancer cells, where different integrin dimers are activated by ECM ligands as these cells encounter multiple distinct microenvironments throughout the body, and thus are switching between active and inactive states, proper trafficking is likely critical to their tumorigenic properties. Previous reports note the possibility of bi-directional control of integrin and growth factor receptor membrane trafficking [37]. Both HER2 and integrin trafficking and recycling regulation may influence the invasive and metastatic potential of breast cancer cells co-expressing these proteins, and could have therapeutic consequences as recycling effects the amount of surface protein available for drug targeting.

### Impact of αv-integrin and HER2 on migration and invasion

HER2 and integrins are both known to influence cell motility [4,30,36]. We demonstrated that deficiency of αv-integrin and/or HER2 can inhibit chemotactic motility in vitro (Fig 5A). While there was a reduced growth rate in clones with low αv-integrin and high HER2 expression (Fig 5B), this does not likely account for the lower number of cells in the migration and invasion assays as the clonal cell line lacking both αv-integrin and HER2 displayed rapid growth and significantly decreased chemotactic motility. Thus, the observed reduction in cell motility was not a passive effect of lower cell numbers, but rather a direct reflection of compromised motility due to a deficiency of αv-integrin and HER2.

When the invasive phenotype of these clonal populations was assessed in vivo, a significant gross morphological change was observed. It is important to assess cell motility in the brain microenvironment as it plays a crucial and multifactorial role in the progression and metastatic dispersal of tumor cells [19]. In the xenografted breast cancer brain metastasis model, the tumor infiltrative growth phenotype was dependent on both αv-integrin and HER2. The αv+H2+ MM2BH cells exhibited a dispersed and infiltrative phenotype in the brain microenvironment, while tumors of clones lacking HER2 and αv-integrin have a compact growth pattern lacking perivascular infiltration. A more infiltrative phenotype would increase the difficulty of achieving full tumor resection in patients, increasing the likelihood of tumor recurrence. As the least infiltrative phenotype observed in our study was in the dual low HER2 and low αv-integrin expressing cells, the combined targeting of αv-integrin and HER2 may have clinical advantage in the resection of solid tumors.

A limitation of this study is the use of a single brain-trophic cell line for most experiments, albeit multiple knockdown clones were studied using 2 independent αv-integrin shRNAs, and we evaluated both in vitro and in vivo growth. Additional studies are required to replicate the experiments in cell lines of different origin to confirm the findings. Intracerebral implantation of tumor cells does not fully mimic the heterogeneity of human brain metastasis localization or progression. Hematogenous spread of brain metastasis will be used in future experiments by intracarotid or intracardiac infusion of tumor cells [15,21].

### Clinical relevance and conclusions

The interactions of αv-integrin and HER2 suggest that expression of αv-integrin may serve as a biomarker for the prognosis of high-HER2 expressing breast cancer. Expression of HER2 in conjunction with αv-integrin induces infiltration of breast cancer cells into brain parenchyma rather than growth as a localized mass. The infiltrative phenotype has implications for surgery. Additionally, diffuse tumor growth may provide a sanctuary for the metastatic cells behind a relatively intact blood brain barrier, which may increase the difficulty of detecting tumor extent and decrease chemotherapy permeability and anti-tumor response [23]. Our result showing the increased cytoplasmic retention of HER2 in the αv-integrin knockdown cells provides a foundation to propose that the expression level of αv-integrin may correlate with the patient’s response to HER2-targeted drugs (such as trastuzumab or Trastuzumab-emtansine immunoconjugate) that requires binding the HER2 receptors on the membrane [1,23]. Combined targeting of αv-integrin and HER2 may provide clinical advantage to the HER2-positive breast cancer patients. In support of this hypothesis, targeting αvβ6 integrin in combination with trastuzumab was effective therapy even in trastuzumab-resistant tumor models [28].

Taken together, our findings highlight the complex integration and regulation of integrin and HER2 signaling on the plasma membrane of neoplastic breast cancer cells. Assessing spacio-temportal attributes of the specific integrin-HER2 protein-protein interaction, and understanding cellular signaling cascades influenced by this interaction are exciting directions for future investigation, and may provide deeper insight into integrin-HER2 coupling for therapeutic intervention.

## Supporting Information

S1 Fig Original Western for Fig 1. (TIF)

S2 Fig Original flow for Fig 2. (TIF)

S3 Fig Original immunoprecipitation for Fig 3. (TIF)

S4 Fig Original flow for Fig 4. (TIF)

S5 Fig Clone 3 histology for Fig 6. (TIF)

S6 Fig Clone 4 histology for Fig 6. (TIF)

S7 Fig Clone 7 histology for Fig 6. (TIF)

S8 Fig Clone 8 histology for Fig 6. (TIF)

S9 Fig Clone 10 histology for Fig 6. (TIF)

S1 Table Data table for Fig 1. (TIF)

S2 Table Data tables for Fig 5. (TIF)

S3 Table Data table for Fig 6. (TIF)
