# Supplementary figures and images for: Interactions between αv-Integrin and HER2 and Their Role in the Invasive Phenotype of Breast Cancer Cells In Vitro and in Rat Brain
Source: PLoS One. 2015 Jul 29;10(7):e0131842. doi: 10.1371/journal.pone.0131842 (PMC4519046; doi:10.1371/journal.pone.0131842)

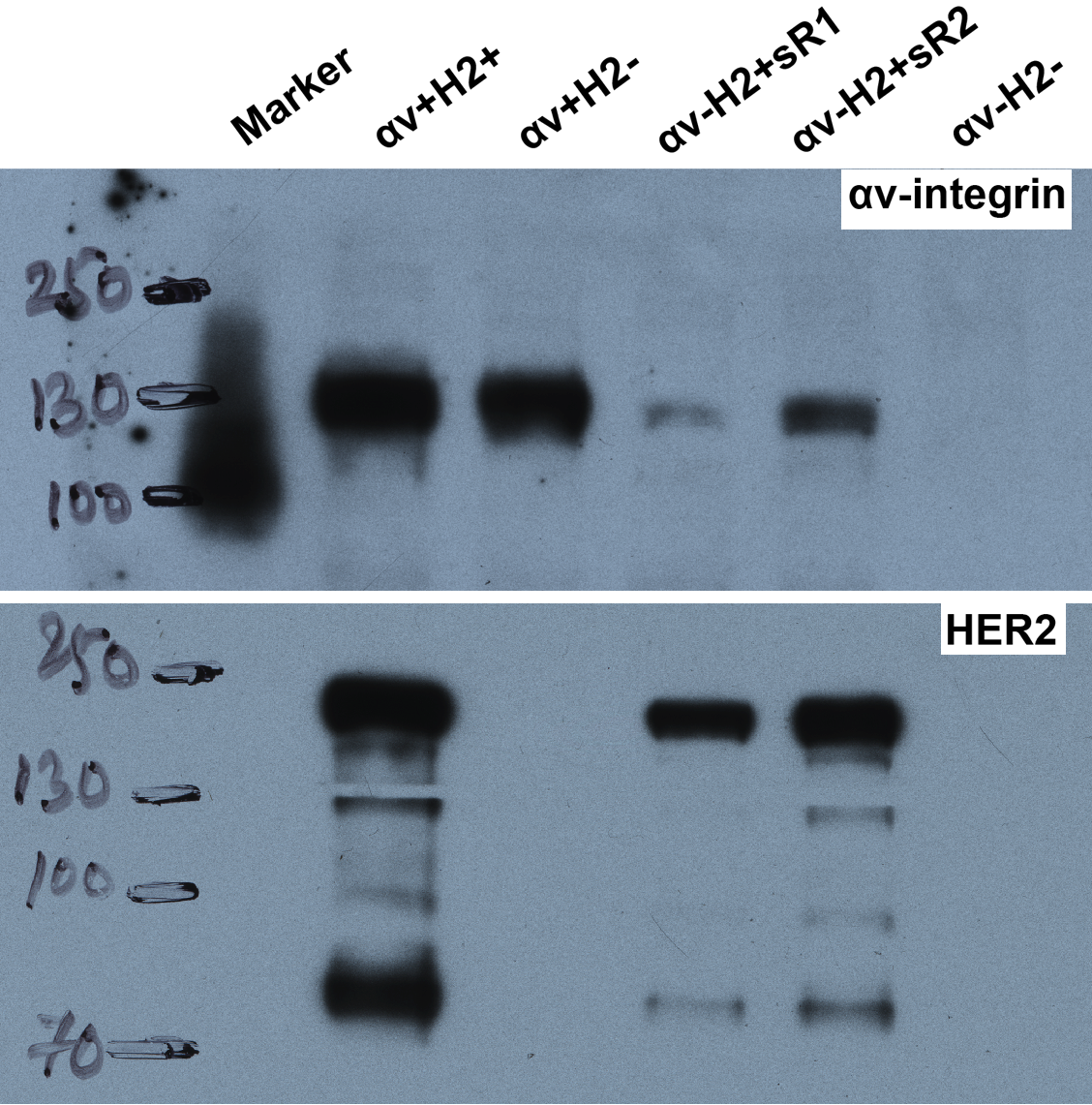

Supplement: S1 Fig — (TIF) [file pone.0131842.s001.tif]

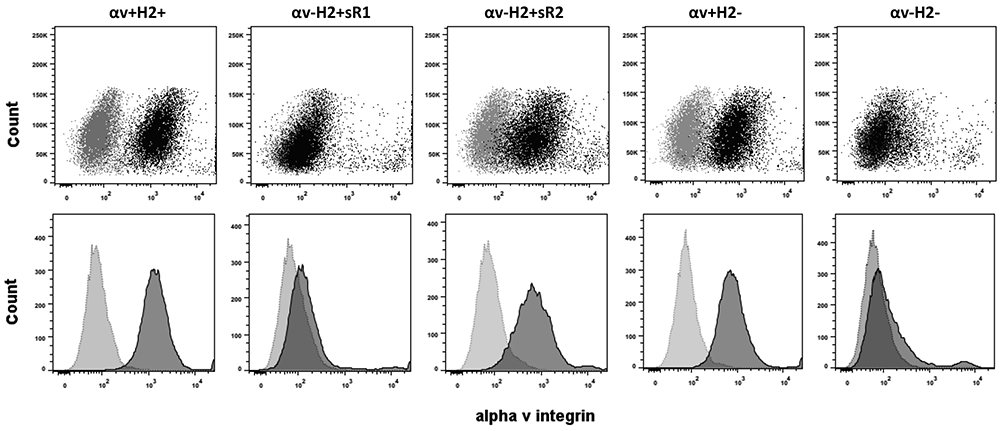

Supplement: S2 Fig — (TIF) [file pone.0131842.s002.tif]

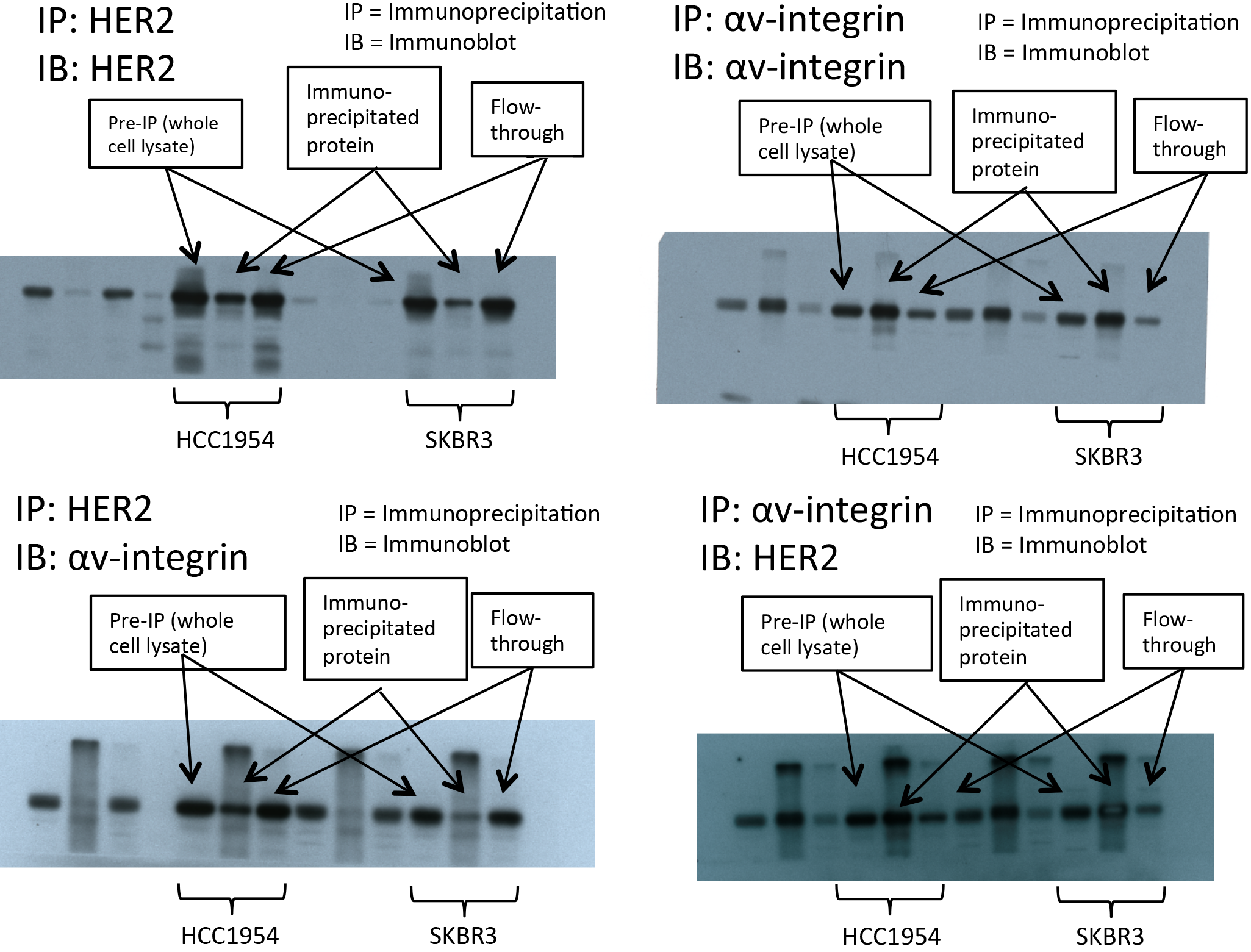

Supplement: S3 Fig — (TIF) [file pone.0131842.s003.tif]

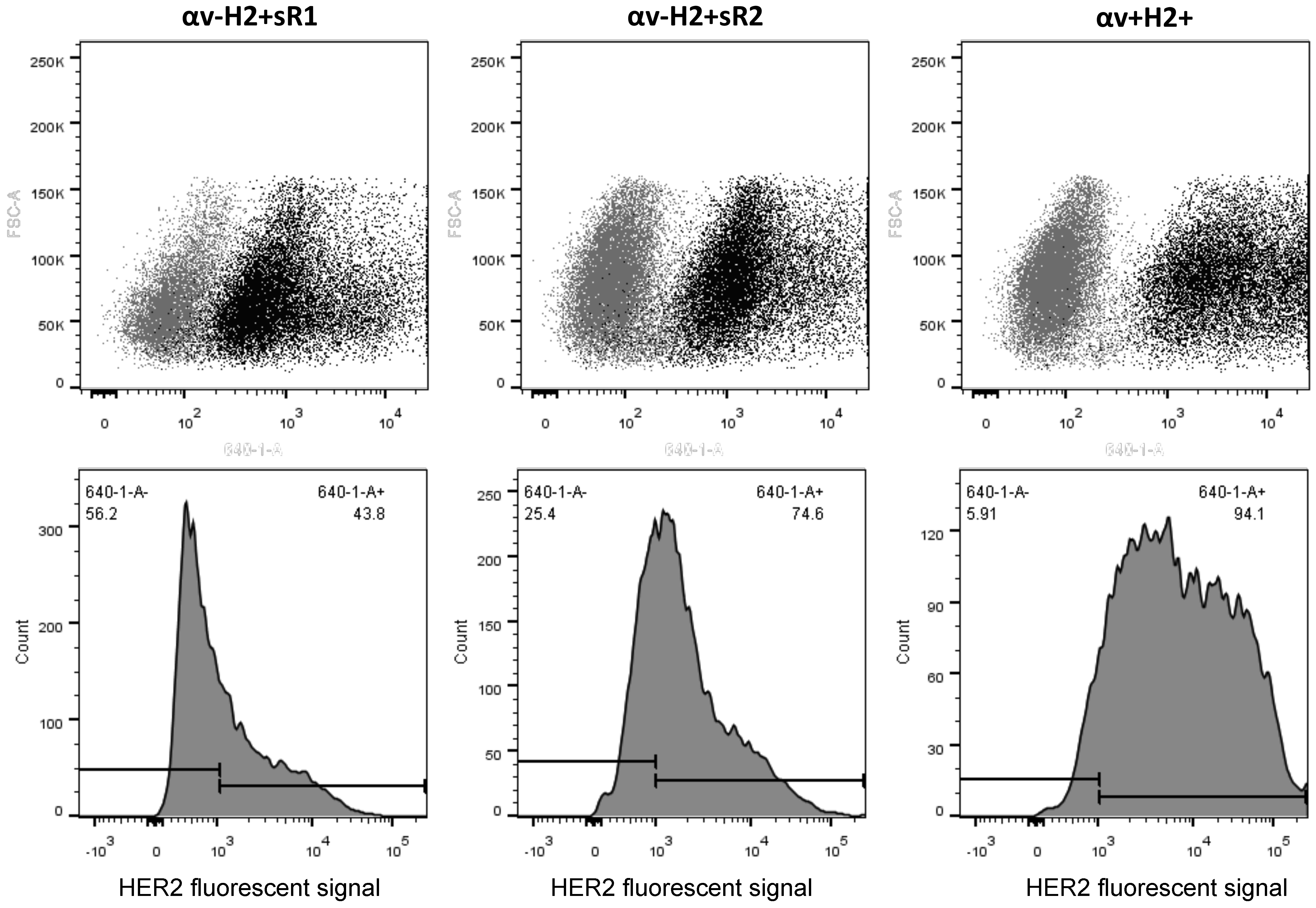

Supplement: S4 Fig — (TIF) [file pone.0131842.s004.tif]

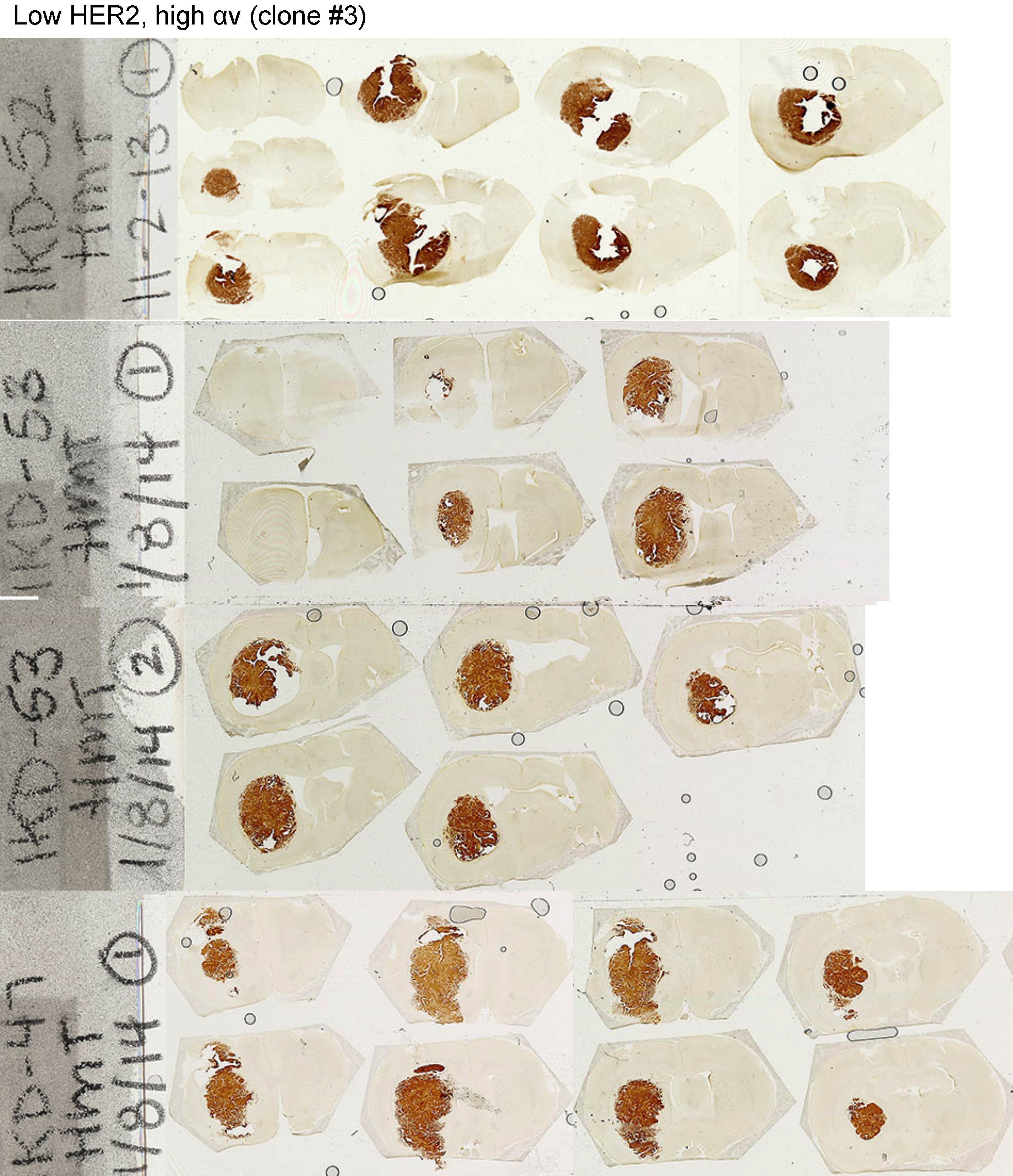

Supplement: S5 Fig — (TIF) [file pone.0131842.s005.tif]

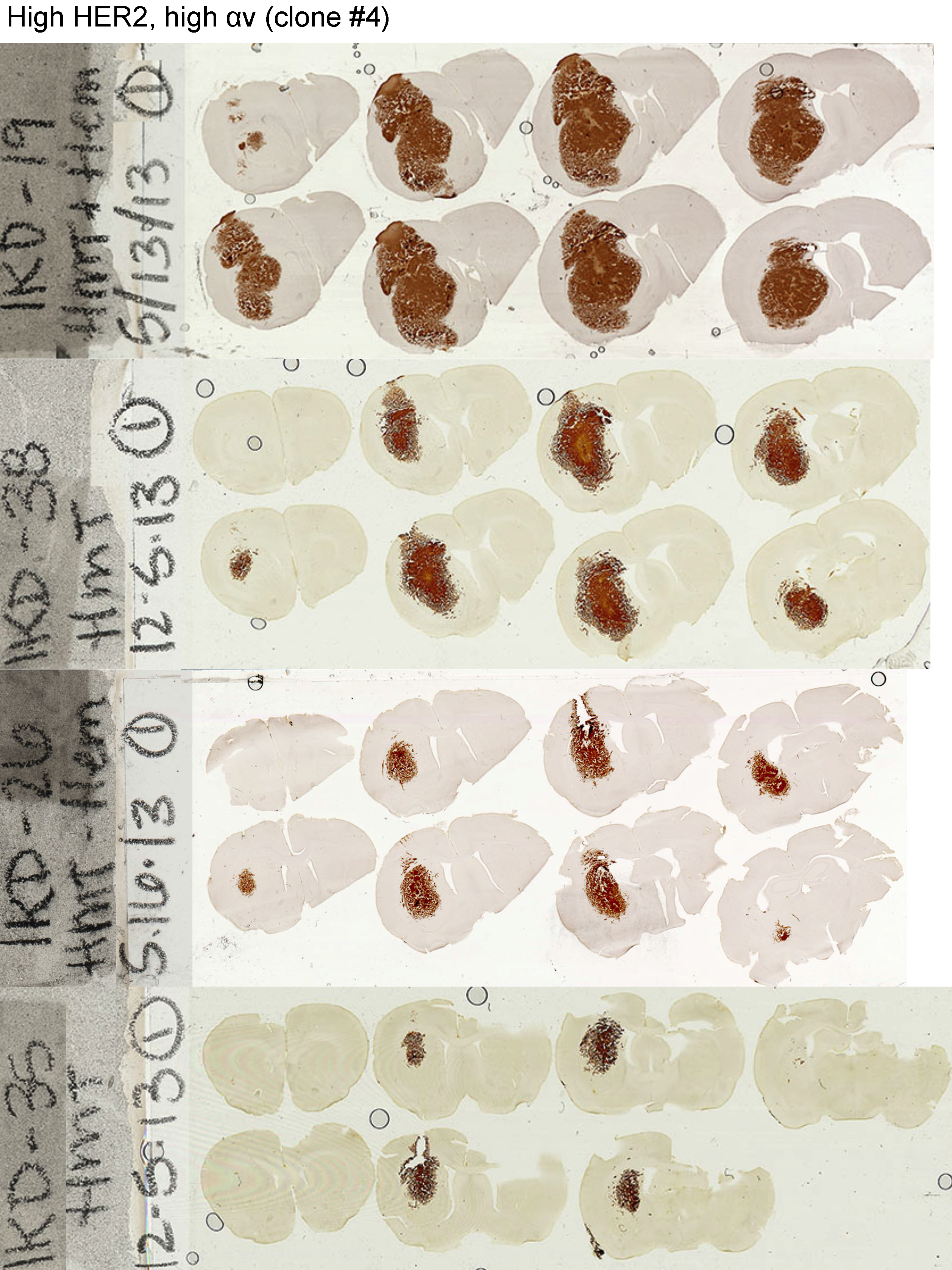

Supplement: S6 Fig — (TIF) [file pone.0131842.s006.tif]

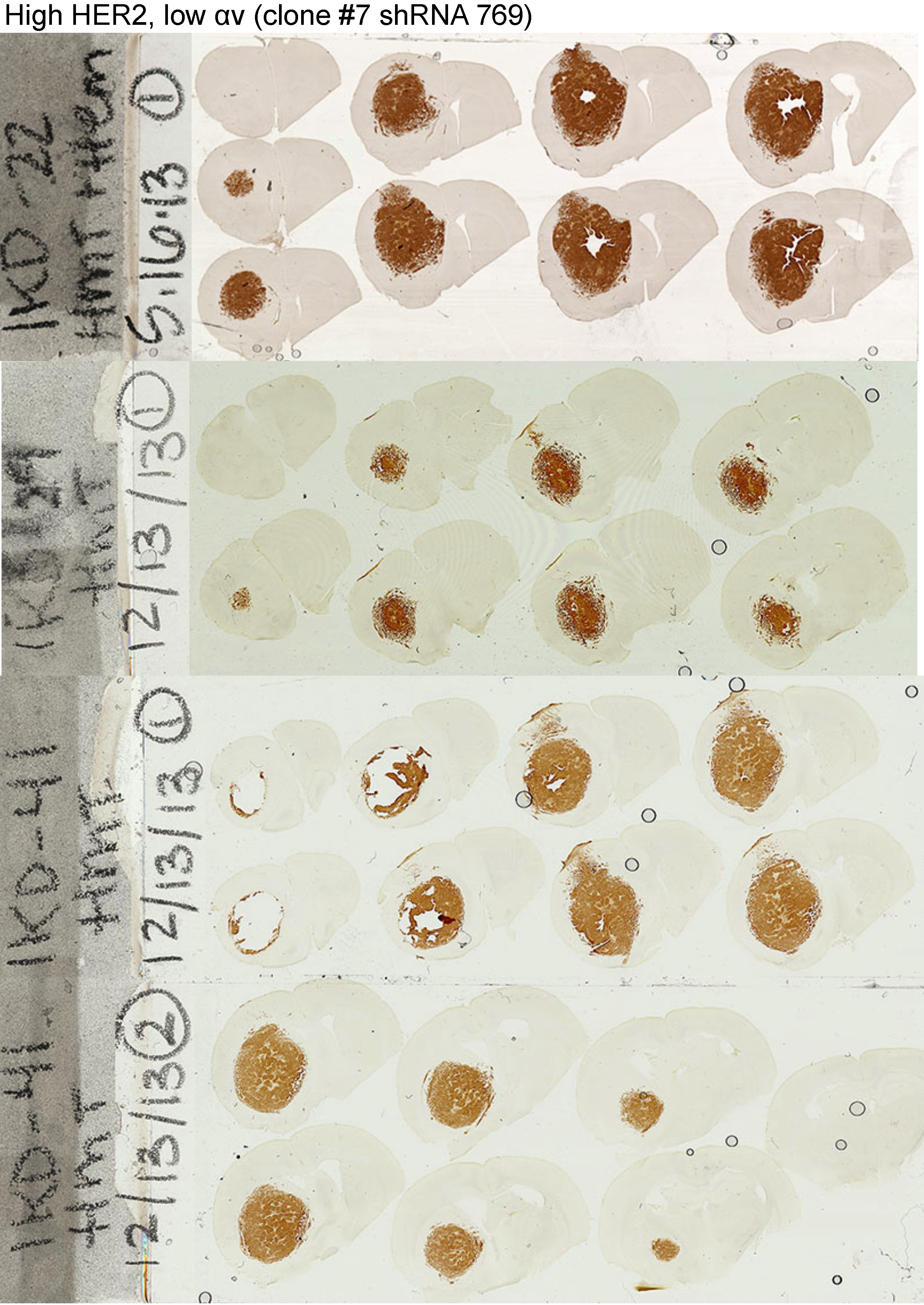

Supplement: S7 Fig — (TIF) [file pone.0131842.s007.tif]

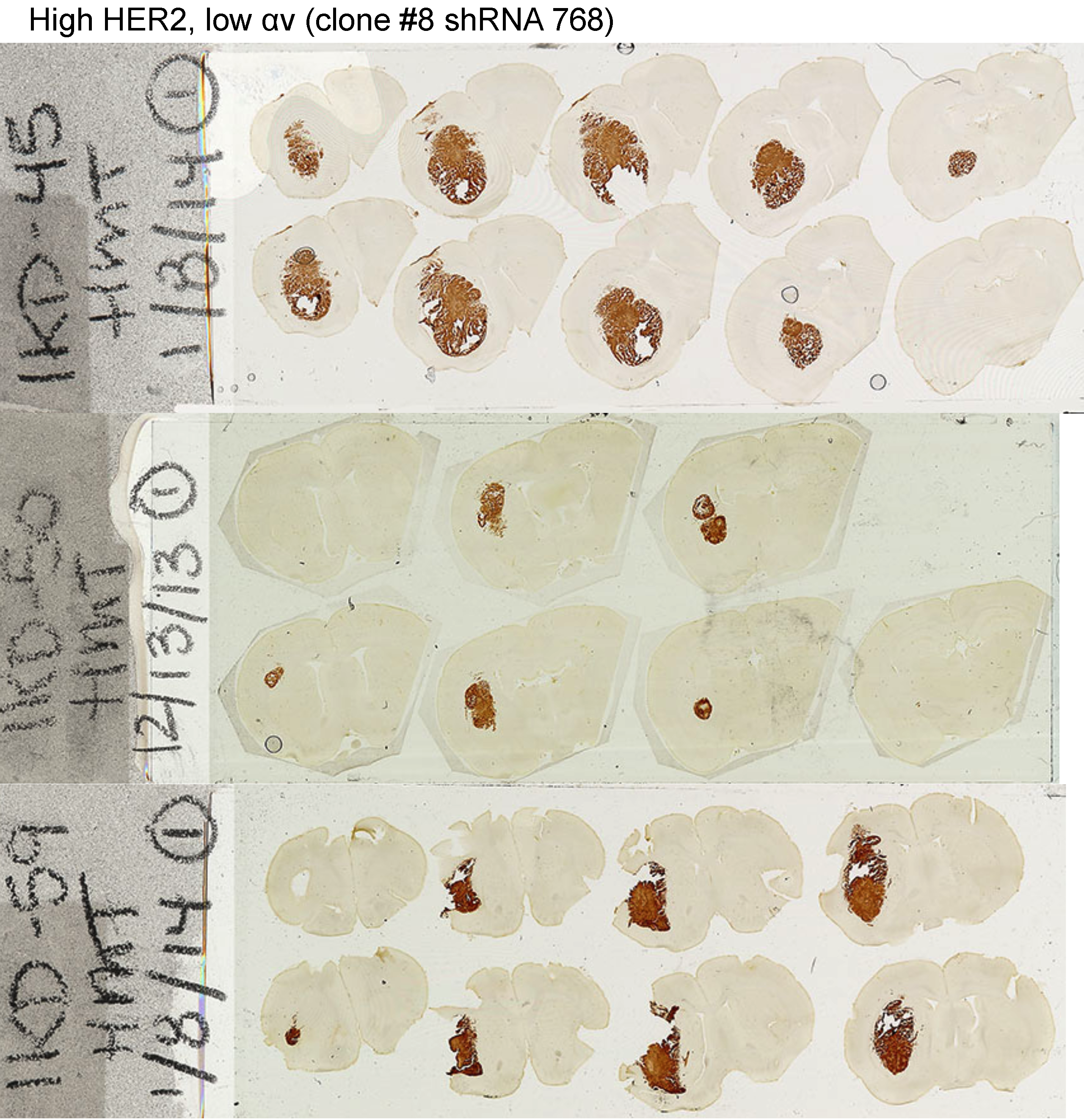

Supplement: S8 Fig — (TIF) [file pone.0131842.s008.tif]

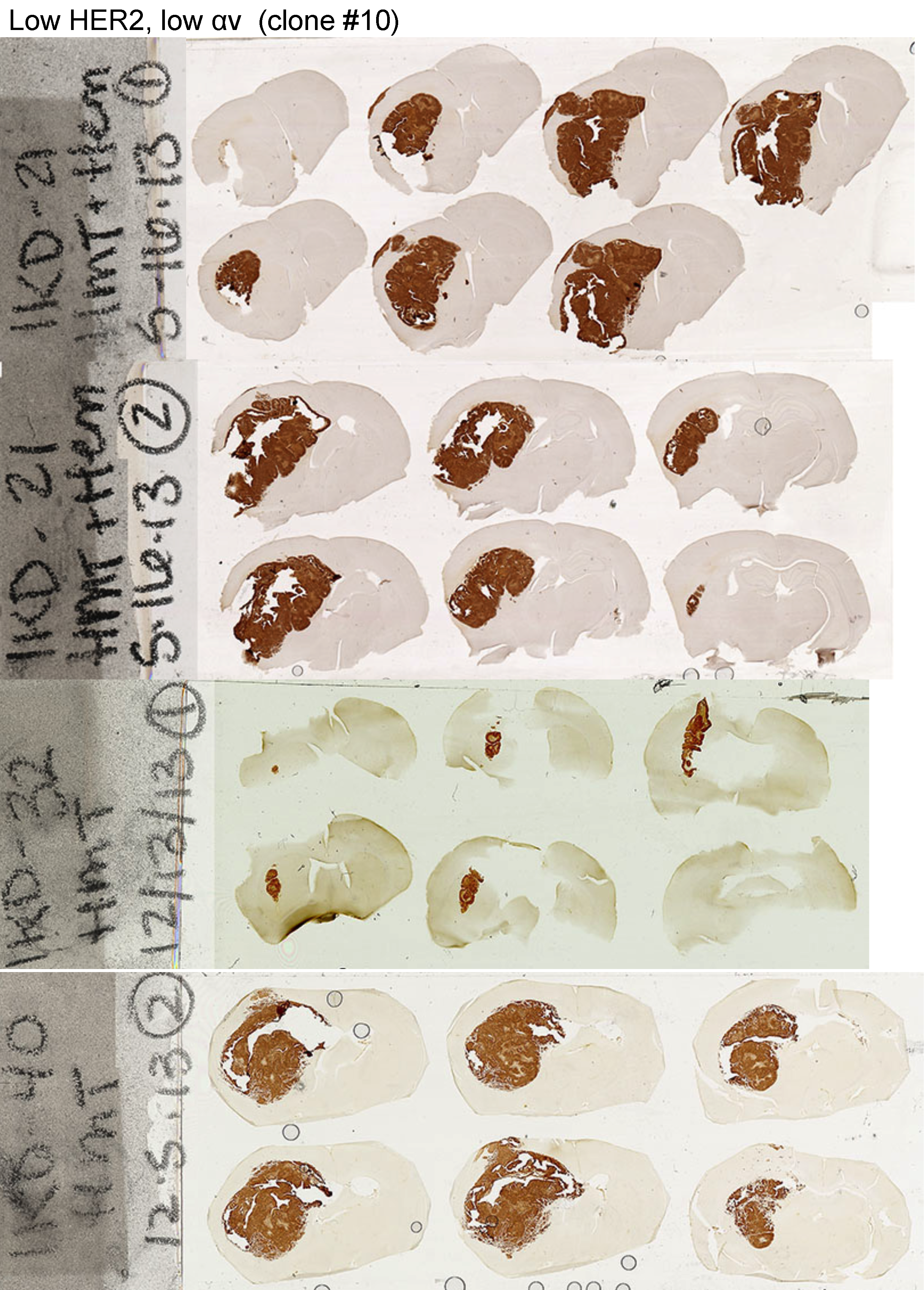

Supplement: S9 Fig — (TIF) [file pone.0131842.s009.tif]

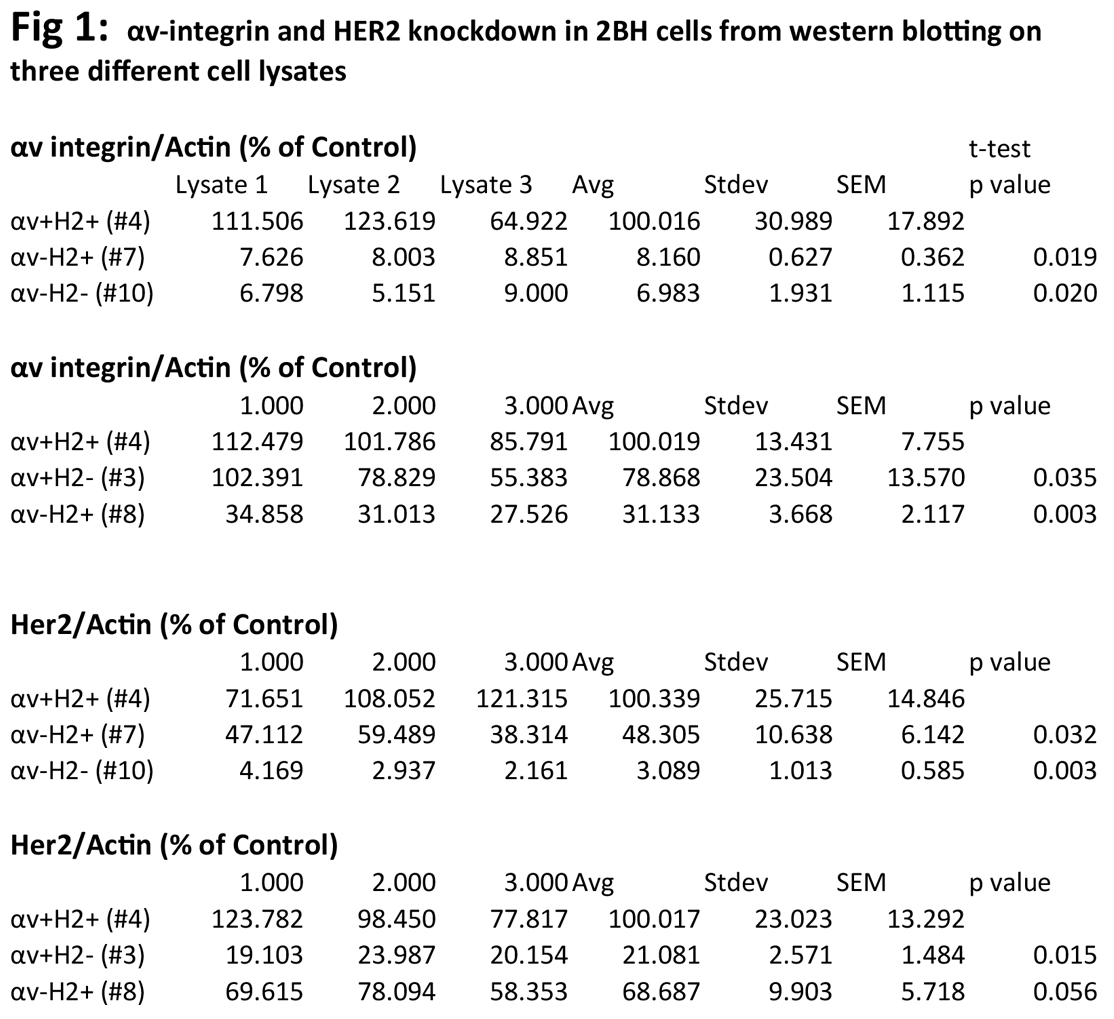

Supplement: S1 Table — (TIF) [file pone.0131842.s010.tif]

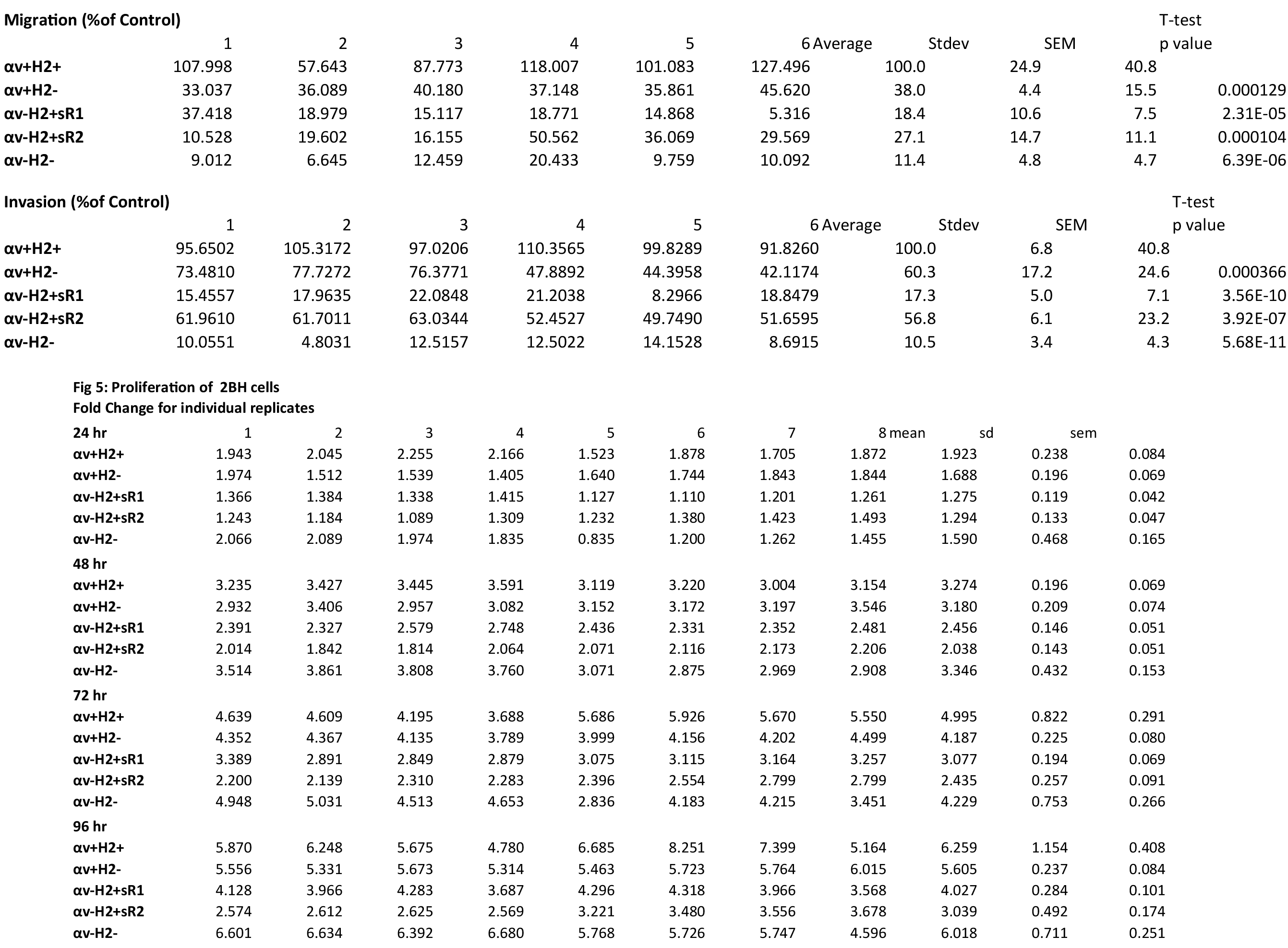

Supplement: S2 Table — (TIF) [file pone.0131842.s011.tif]

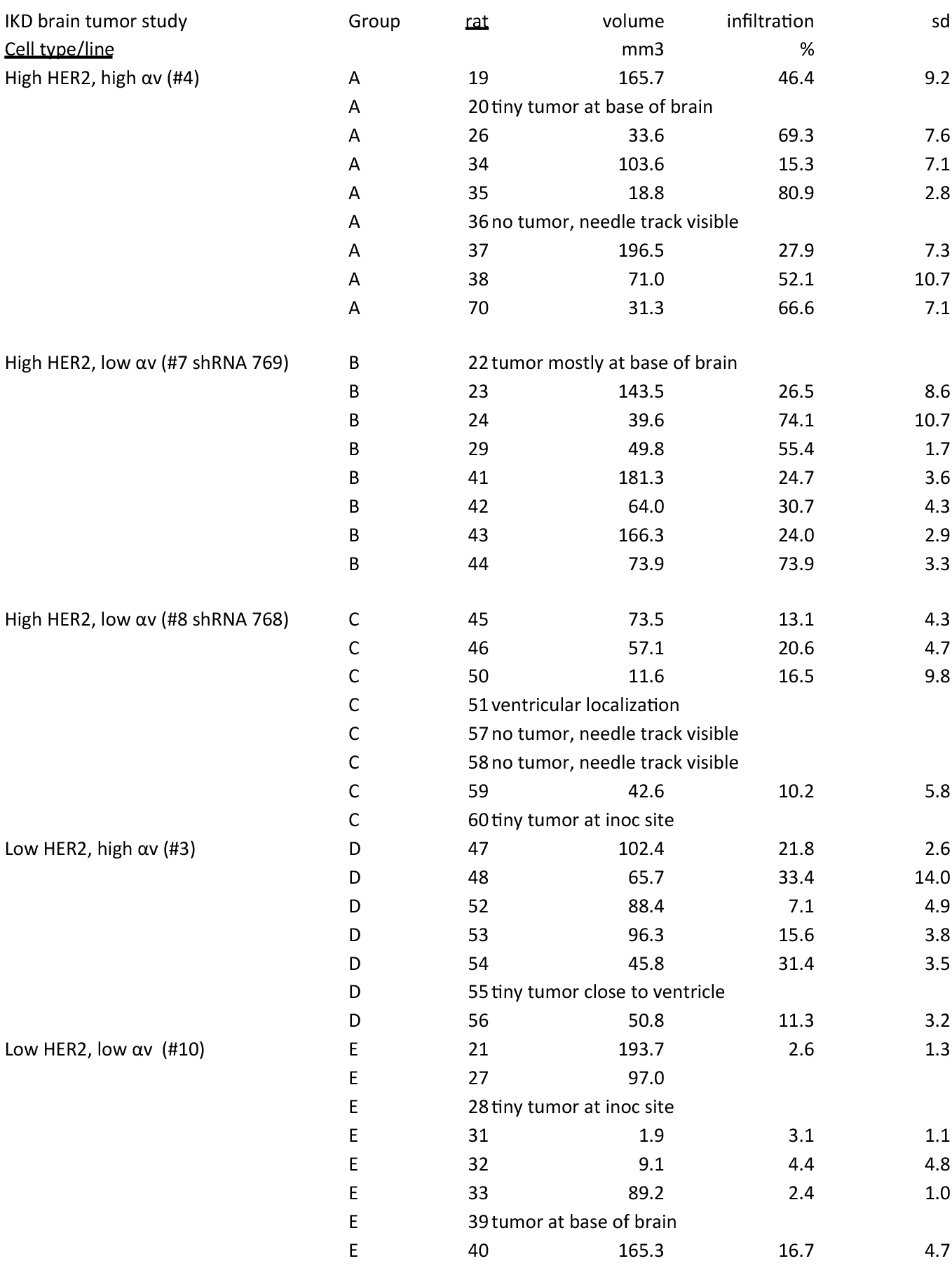

Supplement: S3 Table — (TIF) [file pone.0131842.s012.tif]
